# Supplementary material for: Statistical Distributions of Genome Assemblies Reveal Random Effects in Ancient Viral DNA Reconstructions
Source: Viruses. 2025 Jan 30;17(2):195. doi: 10.3390/v17020195 (PMC11861991; doi:10.3390/v17020195)
Supplement: Supplementary file 1 [file viruses-17-00195-s001.zip › viruses-3410629-supplementary.pdf]

Statistical distributions of genome assemblies reveal the contribution of random effects in ancient viral DNA reconstructions

Supplementary Figures

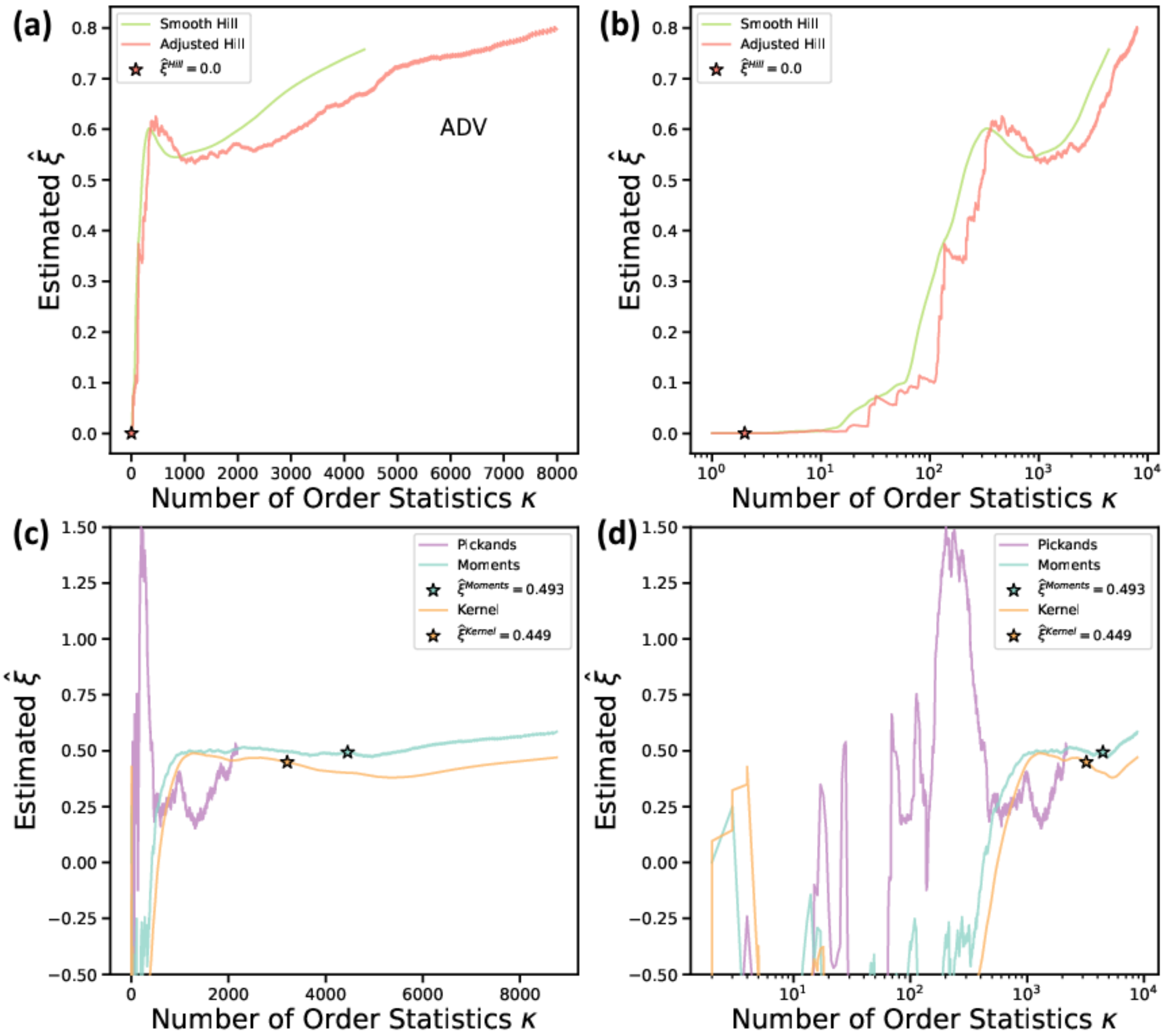

Supplementary Figure S1. ADV assembly. Adjusted Hill (H) estimator and its smoothed version, in original scale (a) and log-scale (b) for the number of simulation steps (number of order statistics *k*). Moments (M) and Kernel (K) estimators in original scale (c) and log-scale (d) for the number of simulation steps (number of order statistics *k*).

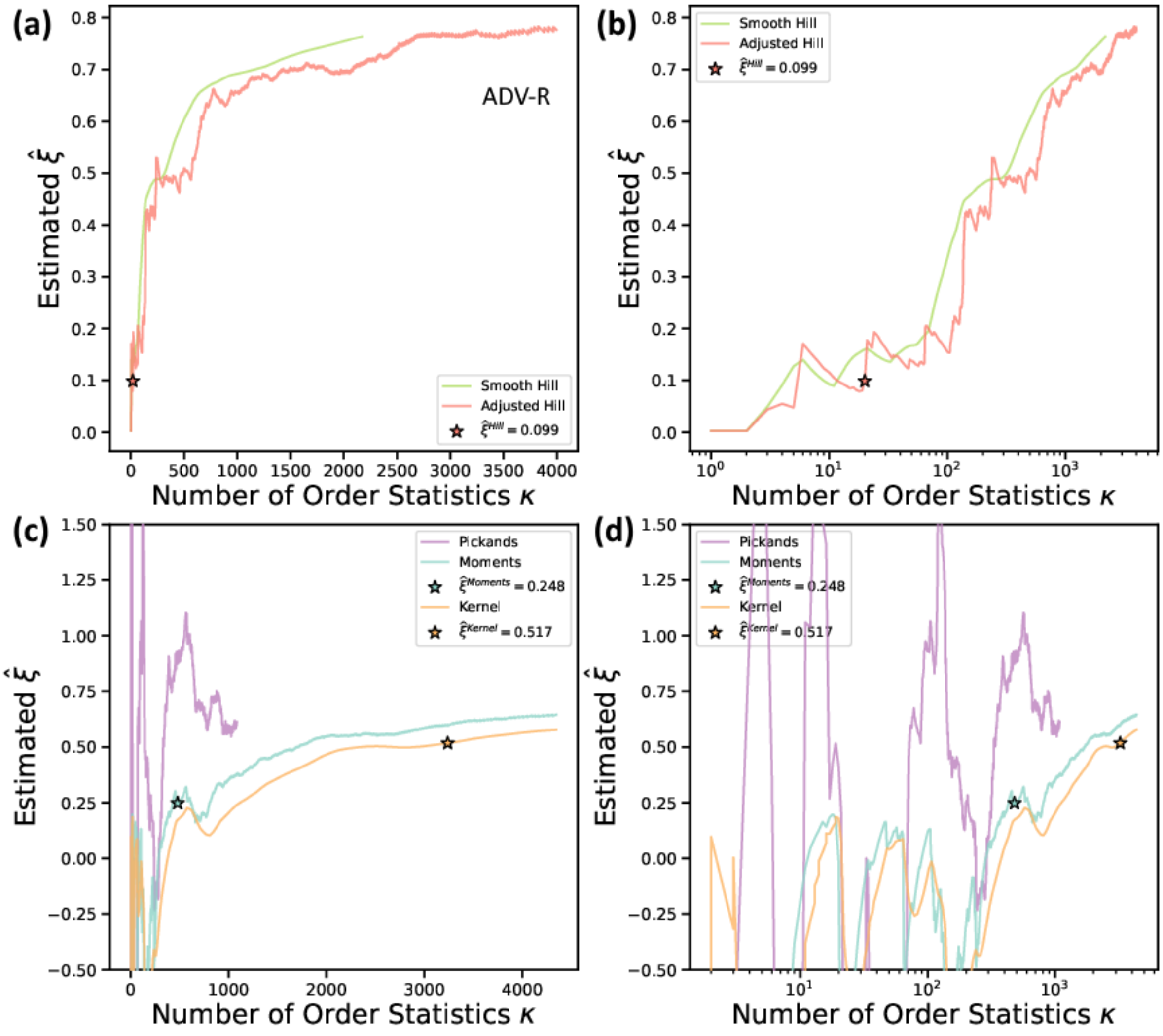

Supplementary Figure S2. ADV-R assembly. Adjusted Hill (H) estimator and its smoothed version, in original scale (a) and log-scale (b) for the number of simulation steps (number of order statistics *k*). Moments (M) and Kernel (K) estimators in original scale (c) and log-scale (d) for the number of simulation steps (number of order statistics *k*).

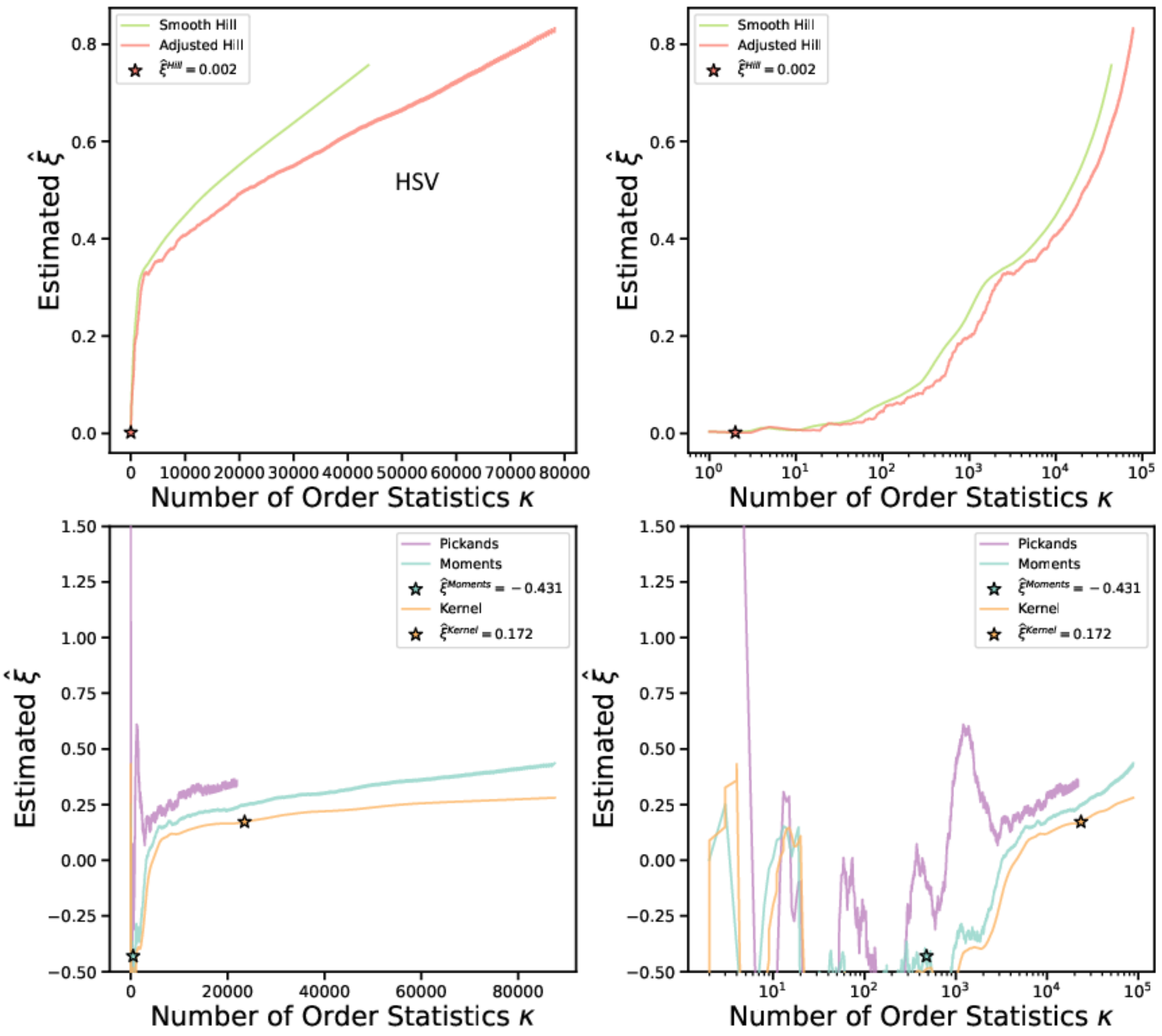

Supplementary Figure S3. HSV assembly. Adjusted Hill (H) estimator and its smoothed version, in original scale (a) and log-scale (b) for the number of simulation steps (number of order statistics k). Moments (M) and Kernel (K) estimators in original scale (c) and log-scale (d) for the number of simulation steps (number of order statistics k).

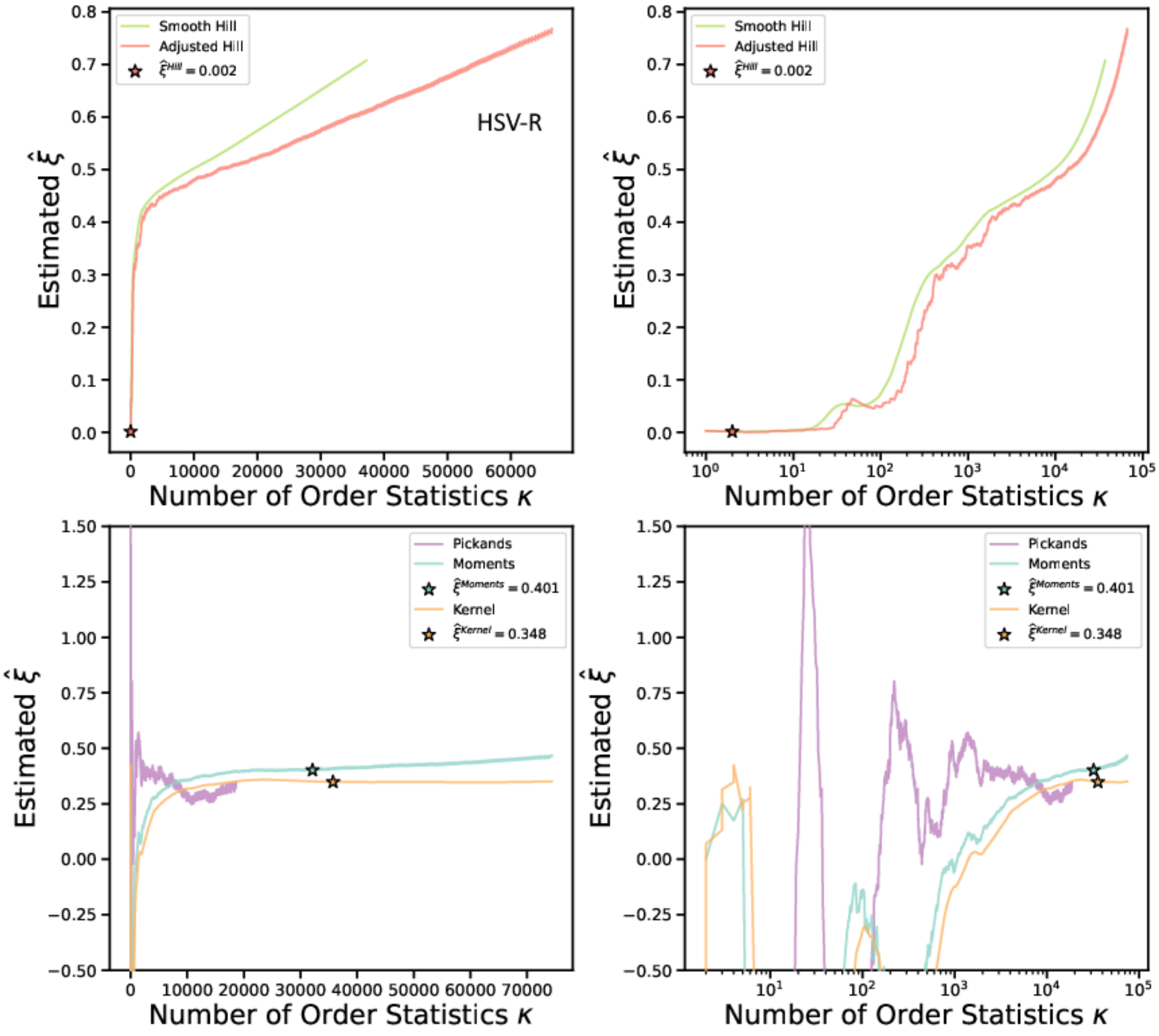

Supplementary Figure S4. HSV-R assembly. Adjusted Hill (H) estimator and its smoothed version, in original scale (a) and log-scale (b) for the number of simulation steps (number of order statistics k). Moments (M) and Kernel (K) estimators in original scale (c) and log-scale (d) for the number of simulation steps (number of order statistics k).

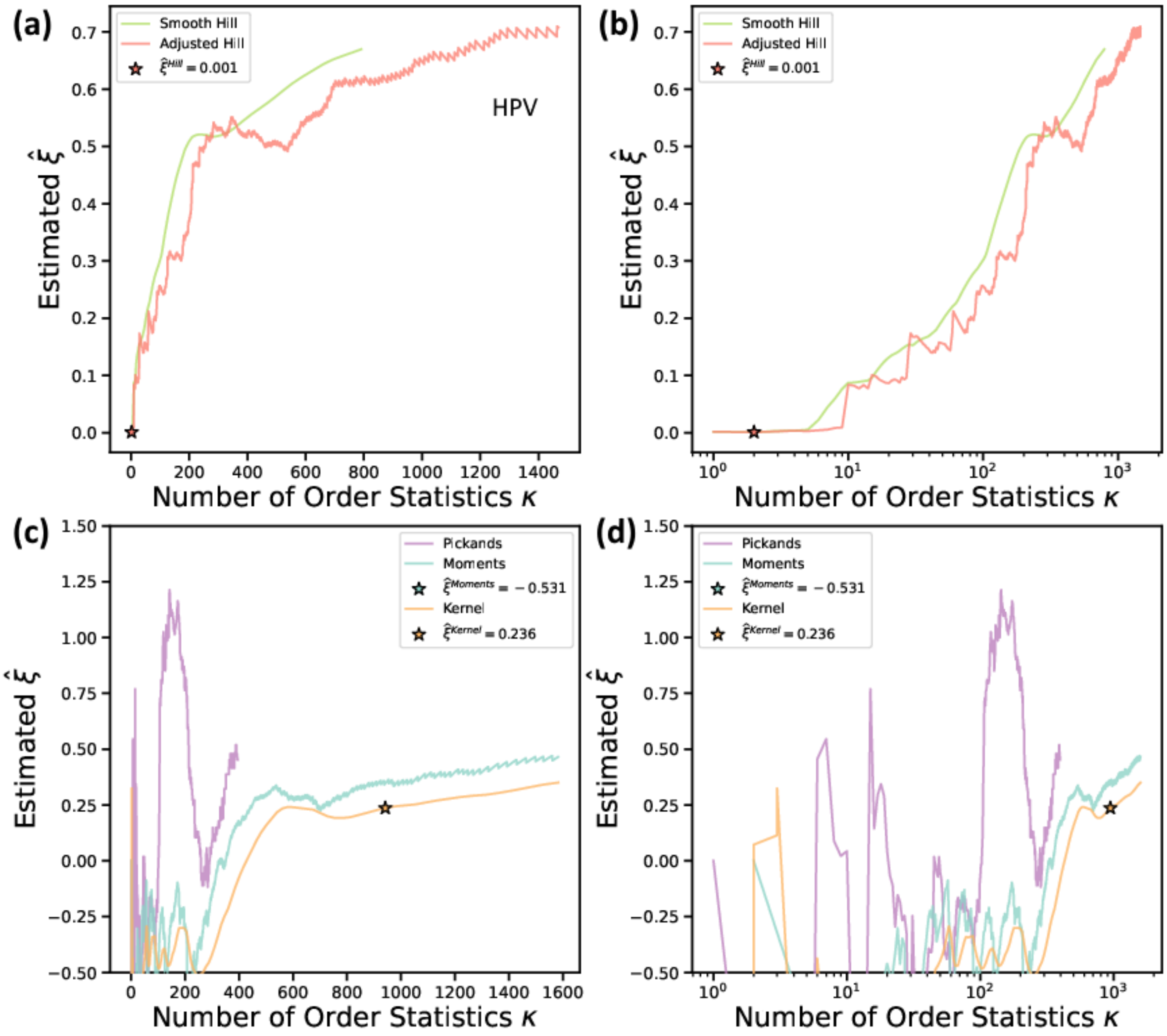

Supplementary Figure S5. HPV assembly. Adjusted Hill (H) estimator and its smoothed version, in original scale (a) and log-scale (b) for the number of simulation steps (number of order statistics *k*). Moments (M) and Kernel (K) estimators in original scale (c) and log-scale (d) for the number of simulation steps (number of order statistics *k*).

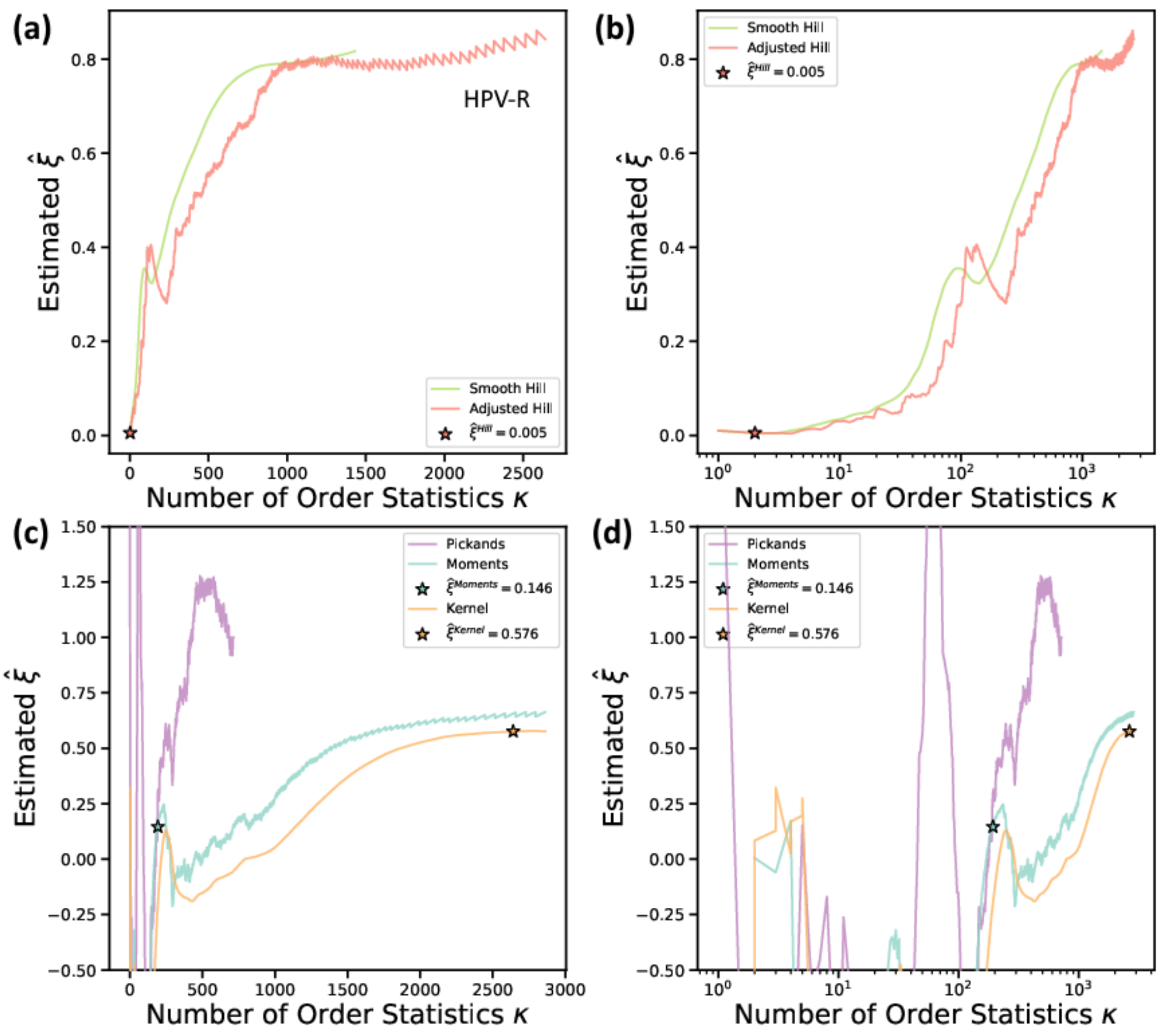

Supplementary Figure S6. HPV-R assembly. Adjusted Hill (H) estimator and its smoothed version, in original scale (a) and log-scale (b) for the number of simulation steps (number of order statistics *k*). Moments (M) and Kernel (K) estimators in original scale (c) and log-scale (d) for the number of simulation steps (number of order statistics *k*).

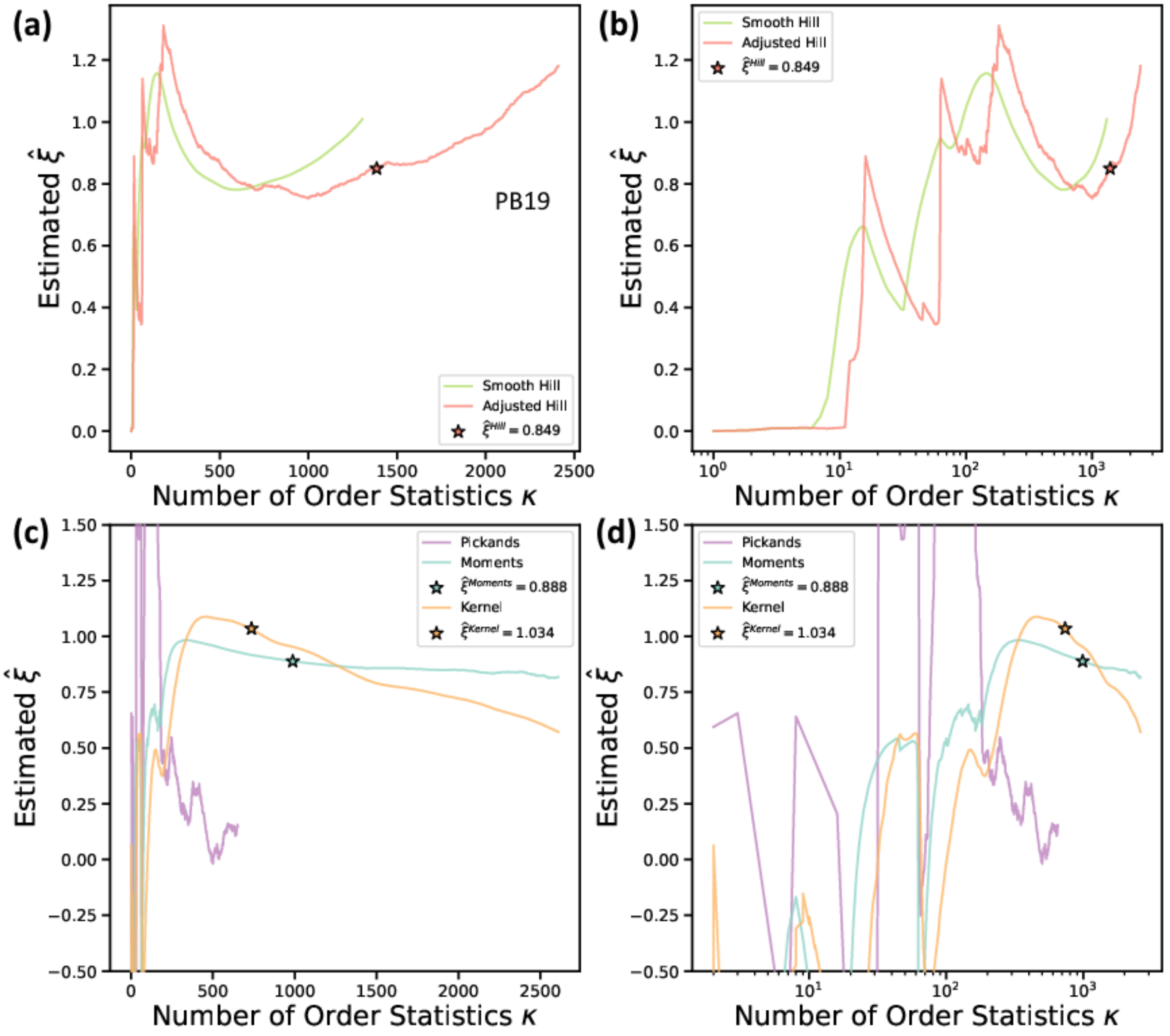

Supplementary Figure S7. PB19 assembly. Adjusted Hill (H) estimator and its smoothed version, in original scale (a) and log-scale (b) for the number of simulation steps (number of order statistics *k*). Moments (M) and Kernel (K) estimators in original scale (c) and log-scale (d) for the number of simulation steps (number of order statistics *k*).

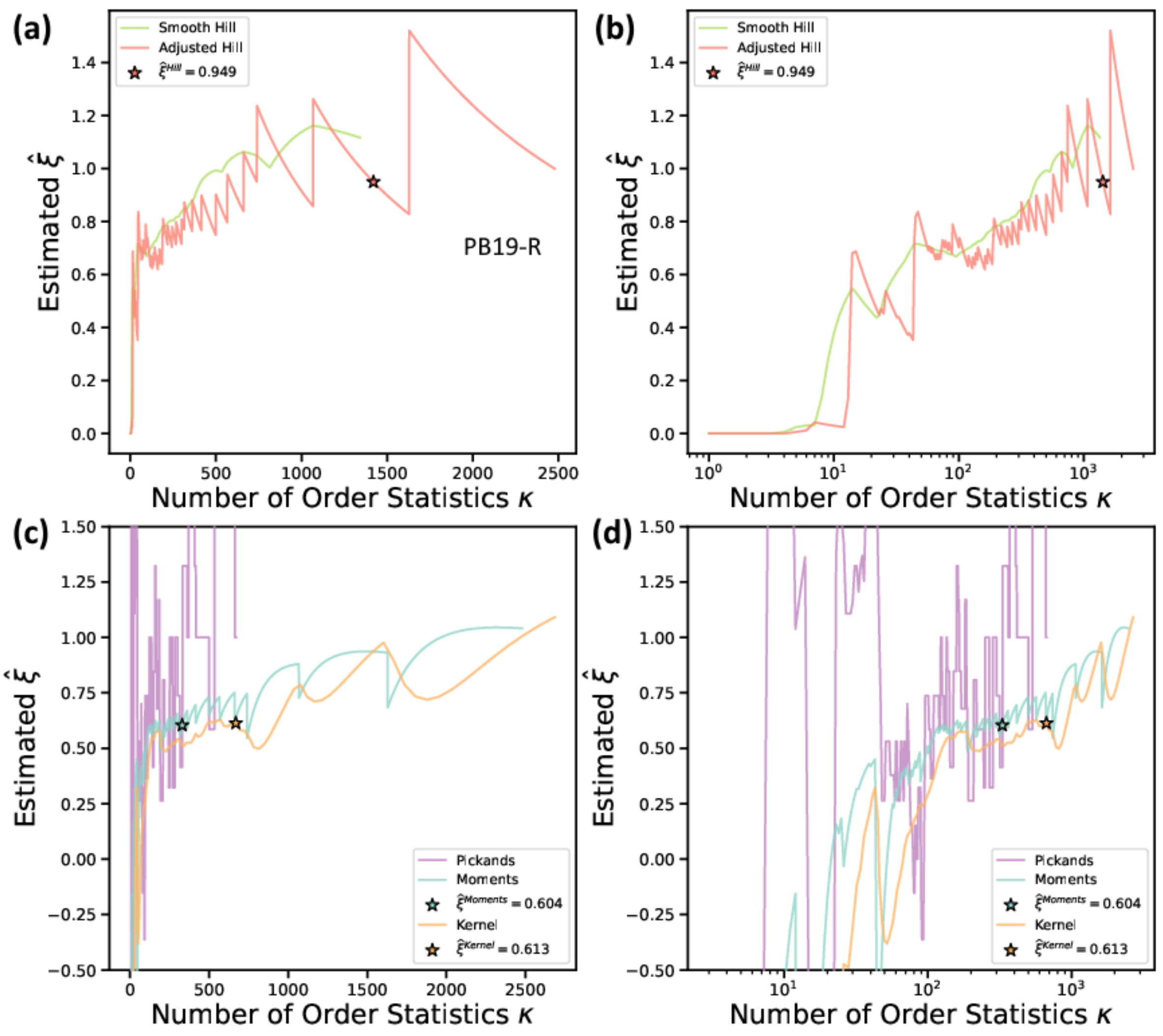

Supplementary Figure S8. PB19-R assembly. Adjusted Hill (H) estimator and its smoothed version, in original scale (a) and log-scale (b) for the number of simulation steps (number of order statistics k). Moments (M) and Kernel (K) estimators in original scale (c) and log-scale (d) for the number of simulation steps (number of order statistics k).
